# The case for addressing primary resistance mutations to non-nucleoside reverse transcriptase inhibitors to treat children born from mothers living with HIV in sub-Saharan Africa

**DOI:** 10.7448/IAS.17.1.18526

**Published:** 2014-01-16

**Authors:** Khady Kébé, Laurent Bélec, Halimatou Diop Ndiaye, Sokhna Bousso Gueye, Abou Abdallah Malick Diouara, Safiétou Ngom, Ndéye Rama Diagne Gueye, Ngagne Mbaye, Haby Signaté Sy, Souleymane Mboup, Coumba Touré Kane

**Affiliations:** 1 Laboratoire de Bactériologie-Virologie, CHU Aristide le Dantec, Dakar, Sénégal; 2 Assistance Publique-Hôpitaux de Paris (AP-HP), Hôpital Européen Georges Pompidou, Laboratoire de Virologie, Paris, France; 3 Faculté de Médecine, Université Paris Descartes, Sorbonne Paris Cité, Paris, France; 4 Hôpital d'Enfants Albert Royer de Dakar, Dakar, Senegal; 5 Centre de Santé Roi Baudouin de Dakar and Synergie pour l'enfant, Dakar, Sénégal

**Keywords:** HIV antiretroviral drug resistance, nevirapine, mother-to-child transmission, children, Senegal

## Abstract

The prevalence of human immunodeficiency virus (HIV) drug resistance mutations (DRMs) was estimated in 25 untreated infants who were living with HIV-1, younger than 13 months and living in Senegal. Antiretroviral DRMs were detected in 8 of 25 (32%) children. Non-nucleoside reverse transcriptase inhibitor (NNRTI) DRMs were present in all (100%) children whose viruses harboured DRMs: K103N in 43%; Y181C, K101E and V106M each in 29%; and Y188L in 14%. The D67N thymidine-analogue mutation was observed in only two children whose mothers had received chemoprophylaxis of mother-to-child transmission (MTCT). The proportion of children whose viruses harboured DRMs was then 6.5-fold higher in children whose mother–child couples had received nevirapine (NVP)-based chemoprophylaxis than in other couples without prophylaxis [7 of 13 (53.8%) vs. 1 of 12 (8.3%)]. These findings point to the absolute need to address primary resistance mutations in case of virological failure in young children treated by antiretroviral drugs, and to make more effective treatment regimens available to NVP-exposed infants living with HIV-1 in Senegal.

## Introduction

According to the Joint United Nations Programme on HIV/AIDS (UNAIDS) estimations, 390,000 children were infected by the human immunodeficiency virus (HIV) in 2011, primarily due to mother-to-child transmission (MTCT) [1]. Indeed, despite the proven effectiveness of chemoprophylaxis for HIV MTCT in clinical trials [2–9], its routine use in the field remains largely challenging [10]. According to the recommendations of the World Health Organization (WHO), nevirapine (NVP) was first introduced for prevention of MTCT, and all further chemoprophylactic regimens contained this highly active non-nucleoside reverse transcriptase inhibitor (NNRTI). The current WHO guidelines (revised in 2010) recommend chemoprophylaxis in pregnant women who are living with HIV and using highly active antiretroviral therapy (HAART) that includes two nucleoside reverse transcriptase inhibitors (NRTIs), such as zidovudine (AZT), lamivudine (3TC) and NVP, through birth and when they are breastfeeding, and a combination of AZT and NVP for children born from mothers living with HIV [11]. Options of prophylaxis and breastfeeding officially adopted in Senegal were option A according to the WHO recommendations until February 2010, followed by option B since April 2010 and option B+ since August 2012. Finally, NVP-based regimens remain the cornerstone of the chemoprophylaxis for HIV MTCT in resource-limited settings [11].

One major pitfall of exposure to NVP is the rapid selection of HIV drug resistance mutations (DRMs) because of its low genetic barrier [12–14], resulting in cross-resistance to both NVP and efavirenz, which constitute the principal NNRTIs of first-line antiretroviral treatment (ART) in most resource-limited settings [15]. Previous studies reported high prevalence of NVP resistance in infants born from mothers living with HIV, reaching for example 40% in South Africa [16] and 87% in Malawi [17]. A recent meta-analysis estimated that the prevalence of NVP resistance in infants six weeks after exposure was as high as 53% [15]. Thus, the selection of antiretroviral drug resistance during the period of chemoprophylaxis for MTCT may compromise further ART and reduce the possibility of choices of active antiretroviral drugs [18].

Finally, the aim of this observational study was to assess the rate of DRMs in untreated Senegalese children living with HIV who were born from mothers living with HIV, including those who have received a NVP-based regimen for vertical transmission prevention, and to discuss their risk of developing therapeutic failure to the first-line WHO ART regimen.

## Materials and methods

### Study children, clinical samples and processing

The microbiology laboratory of Aristide Le Dantec hospital in Dakar, the capital city of Senegal, is a university reference laboratory for HIV monitoring, and it belongs to the national laboratories network. The laboratory is involved in early diagnosis of HIV in children born from mothers living with HIV throughout the whole country.

Blood samples of children born from mothers living with HIV that were sent to the central laboratory for early HIV diagnosis were used for this study, from April 2010 to May 2011. No child was treated by HAART at the time of sampling.

Blood samples consisted of dried blood spots (DBSs) which were prepared by spotting several drops of blood collected from the heel or big toe of the child onto the pre-marked circles on Whatman 903 filter paper cards (Schleicher & Schuell, Whatman, Versailles, France). Briefly, the pre-printed circles were completely filled. After air drying overnight at room temperature (22–37°C), the Whatman filter papers were stored in plastic bags with silica desiccants and a humidity indicator. The bags were sent in an envelope to Le Dantec hospital's microbiology laboratory, where they were kept at −20°C until testing. The Roche Amplicor HIV-1 DNA Test v1.5 was used for qualitative *in vitro* testing for the detection of HIV-1 DNA in whole blood in order to make HIV-1 early infant diagnosis, as described elsewhere [19].

Inclusion criteria were as follows: the children (1) were born from mothers living with HIV-1, irrespective of their immunological and virological monitoring (all mothers living with HIV were exclusively living with HIV-1 not HIV-2); (2) were proven to be living with HIV by virological molecular diagnosis; (3) were never treated by HAART; and (4) had a past history of prevention of MTCT that was available. Exclusion criteria were HIV-1 diagnosis not formally established and lack of information on possible prevention of MTCT.

The study falls within the framework of the national Senegalese ART programme and was formally approved by the Division against HIV/AIDS under the supervision of the Ministry of Health and Social Action. In addition, all of the laboratory investigations were of direct benefit to the children and allowed the orientation of individual therapeutic regimens.

### Antiretroviral drug resistance genotyping and interpretation algorithms

Genotypic resistance tests were carried out. In brief, HIV-1 proviral DNA was extracted from DBS by the NucliSens EasyQ technique (BioMerieux, Lyon, France), amplified to obtain a partial fragment of the reverse transcriptase (RT) of the HIV-1 *pol* gene and further bulk-sequenced using the consensus technique of the Agence Nationale de Recherches sur le SIDA et les Hépatites Virales (ANRS, Paris, France), as described elsewhere [20]. Genotypic resistance profiles were analysed according to the Stanford University HIV drug resistance database (http://hivdb.stanford.edu) and the ANRS-AC11: Resistance group (http://www.hivfrenchresistance.org, version 22).

### Statistical analysis

Fisher's exact test was used to compare proportions, and Graph Pad Prism software version 5.00 was used. The level of significance was set at *p*<0.05.

## Results

### Study population

A total of 25 DBS from untreated children living with HIV-1 who were born from mothers living with HIV-1, corresponding to all inclusion criteria, were prospectively included during the 13-month study period.

Median age was 5.5 months (range: 1.5–17 months), and 16 of the children were male. Breastfeeding was reported by 15 of 25 (60%) of the mothers.

Thirteen children (eight boys and five girls) were infected despite the prevention of MTCT by NVP-based prophylaxis. Mothers principally received option B chemoprophylaxis (maternal triple antiretroviral drug prophylaxis, including NVP), the main regimen (67%) being AZT+3TC+NVP (Table 1), for a mean duration of 60 days (range: 10–252 days). Three mothers were under tritherapy before getting pregnant. Only one mother had received single-dose NVP during labour. Infants received principally option A chemoprophylaxis, including a single-dose NVP or a single-dose NVP plus one week of AZT.

**Table 1:** Antiretroviral drug resistance mutations in viruses of 13 untreated children living with HIV born from mothers living with HIV-1 receiving antiretroviral drug treatment as prophylaxis of mother-to-child HIV transmission, and of 12 untreated children living with HIV born from mothers living with HIV-1 without prophylaxis

Children	Age (months)	Mother prophylaxis	Children prophylaxis	NNRTI resistance mutations	NRTI resistance mutations
1	3	AZT–3TC–NVP	AZT–NVP	**K103N**	**D67N**
2	8	AZT–3TC–NVP	No	**V90I, Y188L**	–
3	3	AZT–3TC–NVP	NVP	**K103N, Y181C**	–
4	1.5	AZT–NVP	AZT–NVP	**K101E, V106M**	–
5	4	AZT–3TC–NVP#	AZT–NVP	**K101E, V106M, P225S**	**D67N**
6	8	D4T–3TC–NVP	AZT–NVP	**Y181C, H221Y**	–
7	3	No	NVP	**K103N**	–
8	3	NVP	AZT–NVP	–	–
9	1.5	AZT–3TC–NVP	NVP	–	–
10	12	AZT–3TC–NVP	AZT–NVP	–	–
11	1.5	AZT–3TC–NVP#	No	–	–
12	5	AZT–3TC–NVP	No	–	–
13	5	D4T–3TC–NVP#	AZT–NVP	–	–
14	3	No	No	–	–
15	8	No	No	–	–
16	3	No	No	–	–
17	1.5	No	No	–	–
18	4	No	No	–	–
19	8	No	No	**K101E**	–
20	3	No	No	–	–
21	3	No	No	–	–
22	1.5	No	No	–	–
23	12	No	No	–	–
24	1.5	No	No	–	–
25	5	No	No	–	–

# Was receiving antiretroviral therapy before getting pregnant. AZT: zidovudine; D4T: stavudine; NNRTI: non-nucleoside reverse transcriptase inhibitor; NRTI: nucleoside reverse transcriptase inhibitor; NVP: nevirapine; 3TC: lamivudine. The bold values correspond to resistance mutations.

The 12 remaining HIV-infected children (8 boys and 4 girls) did not receive any chemoprophylaxis of MTCT. The reasons why NVP chemoprophylaxis was not provided in these latter mother–children couples could not be recorded.

### Genotypic resistance profiles

Antiretroviral DRMs were detected in 8 of 25 (32%) study children (Table 1), including 6 (37%) boys and 2 (21%) girls (not significant). NNRTI resistance mutations were present in all (100%) children whose viruses harboured DRM: K103N in 43%, Y181C, K101E and V106M each in 29%; and Y188L in 14%. In addition, the D67N thymidine-analogue mutation (TAM) was observed in only two children whose mother had received prevention of HIV MTCT. Only one (8%) child born without chemoprophylaxis of MTCT showed virus harbouring the K101E NNRTI resistance mutation.

The mean number of NNRTI resistance mutations was higher in the group of untreated children living with HIV whose mother received a NVP-based antiretroviral drug regimen as prophylaxis than in children whose mother–child couple never received chemoprophylaxis of MTCT (13 mutations of 13 (mean: 1.00 mutation per child) versus 1 mutation of 12 (mean: 0.08 mutation per child); *p*<0.001). The selection of TAM in untreated children was present in 2 of 12 (16%) mother–child couples receiving AZT for the chemoprophylaxis of MTCT. The mean number of TAM resistance mutations was similar in the group of untreated children living with HIV whose mother received a NVP-based antiretroviral drug regimen as prophylaxis compared to children without chemoprophylaxis (2 mutations of 13 (mean: 0.15 mutation per child) versus 0 mutation of 12 (mean: 0 mutation per child); *p*>0.05, not significant). The proportion of untreated children living with HIV whose viruses harboured DRMs was then 6.5-fold higher in those belonging to mother–child couples receiving chemoprophylaxis than in those not receiving prophylaxis (7 of 13 (53.8%) versus 1 of 12 (8.3%); *p*≤0.03).

## Discussion

In this observational study, the DRM prevalence in untreated infants living with HIV who were younger than 13 months, born from infected mothers and living in Senegal was around 32%. These findings point to the need to address primary resistance mutations in case of virological failure in young children treated by antiretroviral drugs and to make more effective treatment regimens available to NVP-exposed infants living with HIV-1. Interestingly, the MTCT prophylaxis as well as breastfeeding practices were highly variable among mother–child couples. Thus, around half of the mother–child couples in this study did not received NVP chemoprophylaxis, reflecting the practical difficulties that are currently encountered to prevent MTCT in the Senegalese public health system. Furthermore, in study couples receiving chemoprophylaxis for MTCT, the option B chemoprophylaxis in mothers (maternal triple antiretroviral drug prophylaxis), including mainly AZT +3TC+NVP, was generally chosen, whereas chemoprophylaxis in children, when present, was generally option A, including the combination of AZT+NVP, and sometimes only NVP, for one week.

The most frequently observed DRMs were resistance mutations to NNRTIs. The level of DRMs in untreated children may have been somehow underestimated, since minority resistant variants cannot be detected with conventional standard population-based genotyping [21, 22]. Nevertheless, our observations likely reflect the wide use of NVP-based chemoprophylaxis for HIV prevention of MTCT in Senegalese delivering women and neonates living with HIV. Untreated children may have been infected with primary resistant HIV strains, whose rate in the paediatric population is associated with previous practices of chemoprophylaxis of HIV MTCT [23, 24]. Our study clearly shows that the proportion (53.8%) of untreated children living with HIV whose viruses harboured DRMs was much (6.5-fold) higher when chemoprophylaxis of MTCT had been received. These findings are consistent with previous studies from Africa reporting that vertically acquired HIV infection is frequently associated with NVP resistance and NNRTI cross-resistance in children exposed to NVP-based chemoprophylaxis during their mothers’ pregnancies or in the perinatal or postnatal periods [15, 25]. Interestingly, the selection of TAM in study children was low despite the frequent use of AZT in chemoprophylaxis in mother or children, confirming that the selection pressure of thymidine-analogue NRTIs is dramatically less potent than that of NNRTIs when used in the prevention of MTCT [25].

The negative consequences of resistance acquired during the chemoprophylaxis of MTCT are a priori particularly important in the paediatric population, since the number of antiretroviral drugs available for paediatric use is very limited in resource-constrained settings. Furthermore, because of the greater overall duration of antiretroviral drug treatment among children living with HIV, the prevention of the emergence of resistance is mandatory in order to preserve future therapeutic options. The high primary DRM prevalence in the present series of Senegalese infants living with HIV-1 has major therapeutic implications concerning the choice of first-line antiretroviral drug in children born from mothers living with HIV who have received a NVP-based regimen for the prevention of MTCT. Indeed, the use of NVP in first-line treatment in study children would be associated with rapid virological failure in nearly one-third of them (32%) due to the pre-existence of NNRTI-resistant variants. Our recent observations, reporting a high level of virological failure associated with DRM selection in Senegalese children receiving a first-line antiretroviral regimen including NNRTIs [26], are consistent with this latter hypothesis. These findings strongly reinforce the need to avoid first-generation NNRTIs and to instead provide protease inhibitors in the first line in children living with HIV who have received NNRTIs for the prevention of MTCT. Taken together, the selection of NNRTI resistance-associated mutations in Senegalese children living with HIV who are under first-line ART is likely associated with the pre-existence of HIV strains harbouring DRMs acquired during MTCT chemoprophylaxis, in addition to other possible factors such as poor adherence.

Despite the small sample size of the present series, these results suggest that DRM prevalence may be significant in the paediatric Senegalese population living with HIV and younger than 18 months. Scarce previous surveys and studies among vertically infected children living with HIV in resource-constrained settings reported moderate DRM prevalence of 5.4% in Maputo, Mozambique [27]; 6.3% in South Africa [23]; 7.6% in Vietnam [28]; 9.8% in Brazil [29] and 13.9% in the Central African Republic [24]. Although genotypic resistance testing is not routinely available in Senegal, our observations point out the necessity to monitor children receiving first-line regimens by plasma viral load to diagnose as early as possible situations of therapeutic failure and to further operate switch to a new therapeutic line. Furthermore, the assessment of the circulation of primary DRMs in the Senegalese paediatric population living with HIV constitutes a priority in Senegal, as recently recommended by the WHO [30].

In conclusion, a high rate of NNRTI resistance mutations was found in the present series of untreated Senegalese children living with HIV-1 and younger than 13 months, likely because of the wide use of NVP-based regimens in chemoprophylaxis of HIV MTCT. These findings accentuate the important needs of early diagnosis of therapeutic failure by optimal biological monitoring in young children treated by antiretroviral drugs according to the WHO recommendations and of making more effective treatment regimens available to NVP-exposed infants living with HIV-1 in resource-limited settings.
